# Multifaceted Impact of SGLT2 Inhibitors in Heart Failure Patients: Exploring Diverse Mechanisms of Action

**DOI:** 10.3390/biomedicines12102314

**Published:** 2024-10-11

**Authors:** Christos Piperis, Anastasios Marathonitis, Artemis Anastasiou, Panagiotis Theofilis, Konstantinos Mourouzis, Alexios Giannakodimos, Elsi Tryfou, Evangelos Oikonomou, Gerasimos Siasos, Dimitris Tousoulis

**Affiliations:** 1 3rd Department of Cardiology, Thoracic Diseases General Hospital “Sotiria”, National and Kapodistrian University of Athens, 11527 Athens, Greece; piperischristos@gmail.com (C.P.); amarathonitis@gmail.com (A.M.); artemisan93@gmail.com (A.A.); mourouzis2005@yahoo.gr (K.M.); alexisgiannak@hotmail.com (A.G.); elsietr@gmail.com (E.T.); boikono@gmail.com (E.O.); ger_sias@hotmail.com (G.S.); 2 1st Department of Cardiology, “Hippokration” General Hospital, National and Kapodistrian University of Athens, 11527 Athens, Greece; panos.theofilis@hotmail.com

**Keywords:** SGLT2 inhibitor, heart failure, pleiotropic effects, chronic kidney disease, inflammation, oxidative stress, autophagy, gut microbiota

## Abstract

Heart failure (HF) is a growing concern due to the aging population and increasing prevalence of comorbidities. Despite advances in treatment, HF remains a significant burden, necessitating novel therapeutic approaches. Sodium–glucose cotransporter 2 inhibitors (SGLT2is) have emerged as a promising treatment option, demonstrating benefits across the entire spectrum of HF, regardless of left ventricular ejection fraction (LVEF). This review explores the multifaceted mechanisms through which SGLT2is exert cardioprotective effects, including modulation of energy metabolism, reduction of oxidative stress, attenuation of inflammation, and promotion of autophagy. SGLT2is shift myocardial energy substrate utilization from carbohydrates to more efficient fatty acids and ketone bodies, enhancing mitochondrial function and reducing insulin resistance. These inhibitors also mitigate oxidative stress by improving mitochondrial biogenesis, reducing reactive oxygen species (ROS) production, and regulating calcium-signaling pathways. Inflammation, a key driver of HF progression, is alleviated through the suppression of proinflammatory cytokines and modulation of immune cell activity. Additionally, SGLT2is promote autophagy, facilitating the clearance of damaged cellular components and preserving myocardial structure and function**.** Beyond their glucose-lowering effects, SGLT2is provide significant benefits in patients with chronic kidney disease (CKD) and HF, reducing the progression of CKD and improving overall survival. The pleiotropic actions of SGLT2is highlight their potential as a cornerstone in HF management. Further research is needed to fully elucidate their mechanisms and optimize their use in clinical practice.

## 1. Introduction

**Figure 1:**
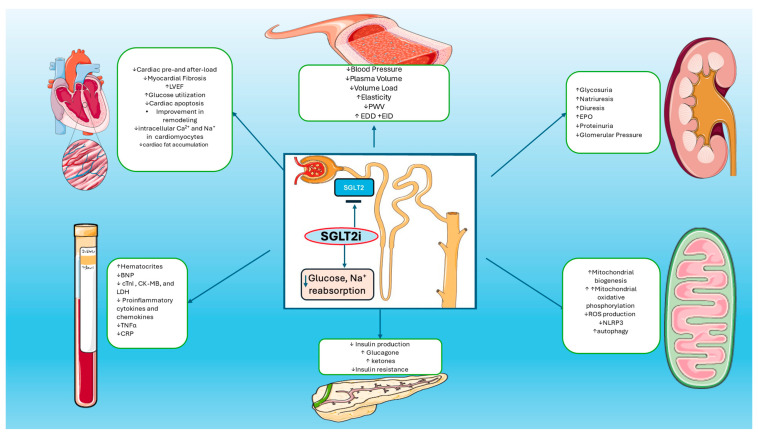
**Mechanisms of Actions of SGLT2is:** SGLT2 inhibitors reduce glucose and sodium reabsorption and promote increased diuresis and glycosuria, plasma volume reduction, and blood pressure reduction. Increased glycosuria leads to reduction in glucose plasma levels and improvement in insulin resistance. SGLT2is also reduce cardiac pre- and after-load, myocardial fibrosis, intracellular calcium in cardiomyocytes, and cardiac fat accumulation. Increased production of erythropoietin leads to the improvement of hematocrit levels. Furthermore, SGLT2is promote reduction in proinflammatory cytokines, TNF-a, and CRP levels. On the other hand, increased mitochondrial numbers and decreased cellular ROS production lead to decreased oxidative damage and enhanced autophagic mechanisms in cardiomyocytes.

Heart failure (HF) prevalence has increased over the last decades. The main reasons are the aging of the population, along with the increasing comorbidities. The lifetime risk of HF is currently at about 20–30% [1]. The incidence of HF in Europe is about 3/1000 person–years (for all age groups) or about 5/1000 person–years in adults [2]. HF treatment is differentiated based on the left ventricular ejection fraction (LVEF). Sodium–glucose cotransporter 2 inhibitors (SGLT2is) are the latest addition to the treatment of HF and the only drug category that has shown benefits in the whole spectrum of HF, regardless of the LVEF [3]. Several studies have been conducted regarding the SGLT2i [4,5,6,7,8,9,10]. These trials have shown beneficial results in patients with HF, with or without diabetes, who already receive the optimal treatment for HF. The beneficial actions of the SGLT2i are observed not only in patients with HF but also in patients with chronic kidney disease (CKD) [11]. The main mechanism of the SGLT2i is the inhibition of the reabsorption of glucose and sodium in the proximal tubule that activates the tubuloglomerular feedback, leads to reduced glomerular hydrostatic pressure, and reduces the GFR (glomerular filtration rate) loss. In addition to that, they have diuretic and natriuretic action which leads to decreased preload and decreased LV filling pressures. Anti-inflammatory actions, reduction in the sympathetic tone, and increase in the hemoglobin levels have been observed too (Figure 1). This evidence supports the idea that the beneficial action of SGLT2i extends far beyond the reduction in glucose levels (Table 1 and Table 2). However, their pleiotropic effects are not fully understood [12,13]. The purpose of this review is to discuss and explain the pleiotropic effects of SGLT2is and their beneficial mechanisms of action in patients with HF.

## 2. Energy Substrate for Myocardial Cells

The human heart must contract incessantly, and so are the requirements for energy. The main form of energy used in human cells is chemical energy in the form of adenosine triphosphate (ATP). In the myocardial cells, the mitochondria occupy about one-third of their volume. In addition, mitochondrial phosphorylation contributes to 95% of the ATP production, and glycolysis provides the remaining 5% [33].

For a healthy heart, the main sources of energy are long-chain fatty acids (60%) and carbohydrates (glucose 30%, lactate 10%). However, there is a minor contribution to the energy production from ketone bodies and amino acids. All these ingredients must be acquired continuously from the blood due to the low ability of the heart to store these energy substrates intracellularly. In terms of energy-producing efficiency, glucose is the most efficient energy substrate, with a glucose phosphate/oxygen ratio (P/0) at about 2.58, compared to a P/O at about 2.33 for the fatty acids and a 2.5 ratio for ketones, which makes ketones the second most efficient source of energy [34]. The contribution of each source alters due to various conditions, either normal such as exercise or diet, or pathological such as diabetes, hypoxia, myocardial ischemia, arrhythmias, and HF. This instant switch between energy substrates is called metabolic flexibility [34]. In these conditions, there seems to be an impairment of metabolic flexibility which leads to suboptimal ATP production. As long as these changes remain short-term, they are well tolerated, and they remain insignificant for the contractility of the myocardial cells [35]. In the failing heart, the metabolic flexibility as well as the mitochondrial function is impaired, mimicking an energy-starved state. More specifically, in HF, impaired mitochondrial function and oxidative capacity result in reduced ATP production by up to 40% compared to the normal heart. The energy substrate is changed from fatty acids to carbohydrates (glycolysis) [36]. Increased expression of GLUT1 seems to be associated with increased glucose uptake in the myocardial cells. Meanwhile, the uncoupling between glycolysis and glucose oxidation, as well as the reduction in the cardiac-branch chain amino acids (BCAAs) catabolism accumulates the glycolytic intermediates as well as the BCAAs. All that accumulation of the BCAAs and the glycolytic intermediates leads to the activation of the mTOR signaling pathway, which is connected to insulin resistance and myocardial remodeling. In addition to that, there seems to be an increasing utilization of ketone bodies as an alternative fuel.

Moreover, SGLT2 inhibitors reduce insulin resistance and enhance peripheral insulin sensitivity. Several mechanisms contribute to these beneficial effects, including the inhibition of glucose toxicity, anti-inflammatory actions, and improvement of oxidative status. These actions lead to a decrease in insulin release, an effect independent of pancreatic alpha and beta cells, as SGLT2 is not expressed in these cells [37,38]. This is a significant factor contributing to heart failure improvement, as insulin, through mitogen-activated protein kinase and its inherent proliferative effects, is implicated in adverse left ventricular remodeling. Additionally, increased insulin levels are associated with sympathetic activation, which can further impair diastolic function [38,39].

Regarding energy substrate HFrEF, the main source is carbohydrates [40,41,42]. On the other hand, HF with a preserved ejection fraction shows a variation in the myocardial energy substrate regarding the underlying cause of HFpEF. In HFpEF caused by diabetes mellitus or obesity, the myocardial FA oxidation increases. However, in HFpEF caused by hypertension or ischemia, it decreases. The commonality in all patients with HFpEF is that the energy–metabolic alteration causes the heart to be less efficient [41,43,44]. Evidence from new studies shows that SGLT2 inhibition mimics a fasting-like metabolic response. With the use of the SGLT2is, the AMPK/SIRT1/PGC-1a pathway is activated, so the energy substrate shifts from carbohydrates to fatty acids and ketone bodies. In addition to that, the activation of the AMPK/SIRT1/PGC-1a pathway is connected to mitochondrial biogenesis [14,15,41]. Moreover, the SGLT2is inhibit the mTOR pathway, and, with that mechanism, they reduce insulin resistance, myocardial fibrosis, and oxidative stress [45]. They also improve cardiac remodeling [14,15].

## 3. Oxidative Stress

The potential modulation of oxidative stress by SGLT2is contributes to cardioprotection, and understanding the complex interactions between oxidative stress and HF progression may redefine treatment strategies.

Oxidative stress precipitates HF through numerous molecular pathways that involve complex cellular interactions. It induces mitochondrial dysfunction, characterized by damage to mitochondrial DNA, proteins, and lipids, as well as disruption of electron transport chain complexes that induce the production of reactive oxygen species (ROS) and mitochondrial permeability transition pore opening. This cascade triggers the release of pro-apoptotic factors, such as cytochrome c, leading to cardiomyocyte apoptosis. Additionally, there are ROS-mediated signaling pathways, such as the activation of redox-sensitive transcription factors like NF-κB and mitogen-activated protein kinases which coordinate inflammatory responses and apoptotic cascades that further exacerbate cardiac dysfunction at the molecular level [46]. Inflammatory pathways triggered by ROS-induced NF-κB activation perpetuate tissue damage through cytokine release and ROS production, forming a self-sustaining inflammatory cycle. Furthermore, ROS-induced DNA damage activates the transcription factor p53, leading to the expression of pro-apoptotic factors such as Bcl-2-associated X protein (BAX), which contributes to cardiomyocyte apoptosis alongside intrinsic apoptotic signaling pathways within mitochondria [47]. Oxidative stress also has profound effects on the electrical remodeling of the heart through redox-sensitive modifications of ion channels, disrupting normal ion flows and promoting arrhythmias and impaired contractility. This could be further elucidated by highlighting the effect of oxidative stress in RyR2 either through direct oxidative modifications or hyperphosphorylation by redox-sensitive protein kinases such as protein kinase A and Ca^2+^/calmodulin-dependent protein kinase II (CaMKII). These processes lead to abnormal calcium handling, predisposing to several arrhythmias as well as contractile dysfunction, and structural remodeling. Furthermore, the ROS-activated CaMKII facilitates the excessive influx of Ca^2+^ by enhancing the late Na+ current. This process results in intracellular Na+ buildup and subsequent Ca^2+^ entry via the Na^+^/Ca^2+^ exchanger (NCX) [48]. Additionally, aberrant Ca^2+^ regulation and contractile dysfunction arise from oxidative stress-induced modifications in the activity of the sarcoplasmic reticulum Ca^2+^-ATPase (SERCA), while ROS also interfere with the establishment of disulfide bridges in cardiac-specific N2B segment of titin, a cytoskeletal that regulates muscle stiffness, leading to myocardial diastolic dysfunction [49,50,51].

SGLT2is have demonstrated promising potential in directly improving cardiac function, through the attenuation of ROS-activated pathophysiological processes. Empagliflozin treatment in animal models intervenes in the cascade that promotes mitochondrial damage by upregulating anti-apoptotic Bcl-2 and downregulating pro-apoptotic BAX. This process prevents mitochondrial outer membrane pore formation and subsequent cytochrome c release, ultimately reducing cellular apoptosis. Additionally, empagliflozin improves mitochondrial biogenesis through the activation of the PGC1α/NRF-1 pathway, leading to increased mitochondrial numbers and decreased cellular ROS production [17].

Furthermore, SGLT2i’s antioxidative properties extend to the facilitation of autophagy, a crucial mechanism for eliminating impaired organelles and proteins associated with oxidative stress. This process is accomplished by inhibiting the mTOR pathway, leading to enhanced autophagic flux and decreased oxidative damage in cardiomyocytes [52].

Additionally, SGLT2is regulate oxidative stress by targeting ion homeostasis and Ca^2+^ signaling pathways within cardiomyocytes. Through the inhibition of the NCX and the enhancement of the sarcoplasmic reticulum (SR) function, these inhibitors re-establish Ca^2+^ homeostasis and reduce ROS production [53]. Additionally, SGLT2is activate AMPK, a key cellular energy sensor. AMPK activation improves mitochondrial function and reduces oxidative stress by regulating ATP production and consumption, thus promoting cellular homeostasis [14,54].

Furthermore, empagliflozin treatment can ameliorate oxidative stress injury in myocardial cells through the inhibition of the transforming growth factor β/Smad pathway and the simultaneous stimulation of the Nrf2/ARE signaling pathway. This process suppresses myocardial fibrosis and upregulates antioxidant enzymes crucial for combating oxidative damage in cardiac tissues [18,55]. Additionally, nicotinamide adenine dinucleotide phosphate 4 suppression and decreased ROS production were established in clinical trials performed in mice treated with either empagliflozin or dapagliflozin [56].

Overall, the multifaceted molecular pathways targeted by SGLT2is collectively alleviate oxidative stress in HF, offering a promising therapeutic approach to improve cardiac function and outcomes in HF patients.

## 4. Inflammation

Inflammation and HF are linked with numerous pathological mechanisms in a vice versa association [57]. A great variety of provocatory mechanisms have been suggested, such as the elevated concentrations of inflammatory cytokines (IL-1b, IL-6, TNF-α), the contribution of pathogen-associated molecular patterns (PAMPs), and damage-associated molecular patterns (DAMPs) pathways, as well as the activation of macrophages and the contribution of other comorbidities in the setting of a chronic inflammatory environment [57,58,59,60]. All these inflammatory pathways lead to endothelial dysfunction, myocardial cell hypertrophy, and fibrosis, leading to systolic and diastolic dysfunction of the heart and finally cardiac remodeling [57].

More specifically, the main axis of the inflammatory path in the setting of HF consists of the innate immune system response through the pattern-recognition receptors, the anti-cardiac antibodies, the proinflammatory monocyte migration from the spleen, and the elevated concentrations of free kappa and lambda chains [61]. The pattern-recognition receptors, such as the Toll-like receptor (TLR)-4 that has the highest expression in the human heart, have a major contribution to the innate immune system. TLR-4 has been associated with myocardial inflammation. Increased concentrations of PAMPs and DAMPs initiate a signaling cascade through the TLR-4, resulting in the NLRP3 inflammasome activation and the expression of IL-6, TNF-a, and NF-κB. In the short term, TLR-4 activation has cardioprotective properties, but in the long-term setting of HFrEF, it seems to drive the recruitment of inflammatory cells and cardiac remodeling [62]. Patients with end-stage HF have anti-cardiac antibodies against the b1-adrenergic receptors, the mitochondrial proteins, the troponin I, the sarcolemmal Na-K ATPase, and the myosin [63]. Furthermore, in chronic inflammatory conditions, there are increased concentrations of free kappa and lambda chains which induce myocardial cell apoptosis and proliferation of cardiac fibroblasts, as shown in animal models [64]. Finally, the migration of proinflammatory monocytes from the spleen infiltrates the heart and leads to the formation of myofibroblasts and interstitial collagen deposition. In addition to the myocardial cell remodeling, the inflammatory process also extends to the endothelium of the coronary vessels. This could be further elucidated by highlighting the deleterious effects, such as the expression of adhesion molecules (Vascular cell adhesion molecule (VCAM)-1, E-selectin), which attract and promote the activation of circulating monocytes. All these pathological mechanisms and pathways finally lead to further interstitial collagen deposition and fibroblast, as well as myofibroblast proliferation. In summary, a chronic inflammatory substrate leads to a fibrotic process resulting in systolic or diastolic myocardial dysfunction [65].

In addition to that, the role of the inflammation extends to the atherosclerosis. The atherosclerotic cardiovascular disease is often expressed as the formation of the atherosclerotic plaques in the coronary arteries. Two types of plaques can be distinguished. The first type is the stable type, which is involved in a reduction in the blood flow in the coronary arteries and often leads to angina. The second type is the vulnerable plaque which leads to acute coronary syndrome and myocardial infarction through its rupture. Atherosclerotic plaques can be found in other arteries as well [66]. At first, endothelial dysfunction occurs under harmful conditions, such as dyslipidemia and hypertension, which result in a chronic inflammatory state. The process begins with the oxidation of the low-density lipoprotein cholesterol (LDL), which is followed by the infiltration of the monocytes in the intimal layer. Oxidated LDL promotes DAMP secretion and a TLR-mediated immune response. In addition to that, the oxidated LDL promotes the expression of adhesion molecules, such as the VCAM-1, and recalls other monocytes and leukocytes. In turn, monocytes transform into activated monocytes and alter the M1 to M2 ratio. The M1 monocytes maintain the chronic inflammation state and the M2 monocytes, as associated with anti-inflammatory properties. In addition to that, the accumulation of the macrophages is associated with the foam cell formation and the activation of the NLRP3 inflammasome, which further promotes atherosclerosis [66,67,68]. The next stage is plaque progression. Extracellular matrix components from the smooth muscle cells contribute to the plaque thickening and growing in the vessel’s lumen. The plaque mostly consists of a central lipid core and a fibrous cap [66]. In this late stage, proinflammatory cytokines and metallopeptidase inhibitors are responsible for plaque erosion. More specifically, the IL-6 induces a prothrombotic state by upregulating the plasminogen inhibitor type 1 and downregulating the protein S and the antithrombin [69].

The SGLT2is have been shown to play a crucial role in this process. In a trial conducted in mice, dapagliflozin attenuated the activation of the NLRP-3 inflammasome and fibrosis [19]. Data from human studies have shown that the SGLT2i reduces the expression of circulating inflammatory molecules such as TNF-α, IL-1, IL-6, intercellular adhesion molecule 1 (ICAM-1), VCAM-1, and the AMPK-dependent pathway [20,70,71]. In addition, human-umbilical-vein endothelial cells that have been exposed to dapagliflozin have shown reduced levels of lipopolysaccharide-induced TLR-4 and the NF-kB expression of p65 phosphorylation [70]. Furthermore, dapagliflozin seems to shift from inflammatory M1 macrophages to M2 dominant macrophages, which suggests that the use of the SGLT2is could demonstrate direct anti-inflammatory properties regardless of the glucose control by activating the NF-κB and by inhibiting the expression of the TLR-4 [70]. Finally, the CANOSSA trial demonstrated that the use of canagliflozin may decrease concentrations of high-sensitive CRP after 3, 6, and 12 months compared to the baseline [72].

## 5. Autophagy

A perfect balance between synthesis and degradation is required to maintain the regular function of eukaryotic cells. This major degradative process is called autophagy or autophagocytosis. This process plays an important role in the decomposition and utilization of damaged organelles and misfolded proteins [73,74]. Autophagy can be categorized into three subclasses: micro-autophagy, macro-autophagy, and chaperone-mediated. From these three categories, macro-autophagy has earned some interest because it seems to play a significant role in patients with HF. Macro-autophagy is initiated by the creation of phagophores. A phagophore is a transient double-membrane structure that is responsible for the isolation of the cytoplasm and the formation of autophagosomes by their fusion with the lysosomes. With that mechanism, the cells are provided with nutrients, and, at the same time, they remove their damaged parts, proteins as well as invading microorganisms. Autophagy-related proteins control this mechanism. In addition to that, the mTOR complex 1 and mTOR complex 2 regulate the process by activation or deactivation [74,75].

Myocardial cells have a limited capability to regenerate or replace damaged tissue. With autophagy, the damaged parts of the cell become a source of energy in the form of ATP to maintain the survival of the cell. At low or moderate levels of stress, the autophagic flux demonstrates its cardioprotective properties. Many sources of myocardial stress seem to activate it, such as hypoxia, cardiotoxic effects, and even ischemia. However, in spite of the fact that these types of stress induce a cardioprotective action through autophagocytosis, they can also cause myocardial death through the autophagy and with the autophagy.

The myocardial cell death with autophagy is called autosis and indicates that excessive autophagic activity is the mechanism that causes the death of these cells. This is observed in reperfusion ischemia and can be prevented by reducing the autophagic flux. In contrast, cardiotoxic effects such as drugs (i.e., doxorubicin) can promote the autophagic process in order to protect the cells. In extreme stress conditions, they fail, and the cell dies [76,77]. In humans, the mutation of the lysosomal-associated membrane 2 protein leads to cardiomyopathy known as “Danon disease” [78]. In addition to that, studies performed on animals show that dysfunction of the autophagy-related proteins and, more specifically, Atg5 protein genes lead to collapsed mitochondria and dysfunctional sarcomeres and eventually to systolic dysfunction of the myocardial cells [79].

Growing evidence supports the idea that the SGLT2is promote the autophagic flux in the myocardial cells as well as in the kidney. In addition to that, SGTL2is accelerate the disposal of the injured mitochondria and restore the healthy mitochondrial function [21]. With their pleiotropic actions, the SGLT2is prevent cell loss by apoptosis and maintain a normal tissue architecture by decreasing inflammation and the fibrotic process. This effect is caused by the increase in the expression of AMPK and the decreased activation of the mTOR protein complex in tissues under stress [76].

Empagliflozin treatment in mouse hearts exposed to doxorubicin is associated with a direct effect on the myocardium and is linked with increased biomarkers of the autophagic flux (increased LC3-II to LC3-I ratio), as well as with an increase at the autophagosomes and autolysosomes and an increased formation of Beclin 1-TLR 9—SIRT3 complex [80].

Furthermore, canagliflozin treatment in kidneys and human HK2 proximal tubule cells leads to increased autophagic flux biomarkers as well through a direct cellular effect before and after inhibiting autophagy with chloroquine. They also verified that Canagliflozin induces the phosphorylation of the AMPK and the dephosphorylation of mTOR and provides protection through the activation of the AMPK [81].

Moreover, dapagliflozin treatment in mouse cardiomyocytes with an ischemia–reperfusion model led to increased autophagic flux biomarkers before and after inhibiting them with chloroquine independently of its hypoglycemic effect and acts directly in the myocardial cells through the NHE 1/NCX pathway [22].

In conclusion, the SGLT2is ameliorate the cell loss, and they provoke the autophagic flux through direct actions in the myocardial cells, as well as with indirect actions through their hypoglycemic effect. Several animal trials have shown that the use of the SGTL-2i is linked with an increased concentration of the autophagic flux biomarkers. Even though more research is needed in that field, recent data emphasize the cardioprotective effects of SGLT2is through regulation of autophagy.

## 6. Kidney Function

CKD is a common clinical condition in which the glomerular filtration rate (GFR) gradually decreases over time. CKD is linked to many clinical diseases such as diabetes mellitus, HF, hypertension, and others. Even though many medical treatments have been linked to the reduction in the risk of CKD progression, so far, no treatments have been shown to prevent the development of end-stage CKD and prolong the survival of the patients. However, new clinical evidence suggests that the use of the SGLT2i in patients with CKD not only decreases the progression of the disease itself but also decreases the progression to dialysis, kidney transplantation, or death due to CKD [82].

At first, the SGLT2is were designed to act as a glucose-lowering agent. Their main mechanism of action is the inhibition of the SGLT in the S1 and S2 portions of the proximal tubule. With this mechanism, higher concentrations of sodium are delivered to the macula densa and stimulate adenosine release, which induces vasoconstriction of the afferent arterioles and vasodilation of the efferent arterioles. This effect reduces intraglomerular pressure and restores the tubuloglomerular feedback, acting as an initial dip in the GFR and reducing the albuminuria in the short term but preserves the kidney function in the long term [83]. In addition, diuresis and natriuresis act beneficial to kidney function as well. Furthermore, the SGLT2is lead to increased glucose concentrations in subsequent tubular segments, which stimulates the production of erythropoietin (EPO). The increased production of EPO is induced by the increased activity of the SGLT2, which decreases the oxygen tension in the outer medulla [84]. A potential mechanism of decreasing proteinuria with a direct effect on the podocytes is described as well [85]. Many indirect effects are mentioned as well, such as the reduction in body fat, the reduction in oxidative stress and inflammation, and the metabolic flux [86,87,88].

Many clinical trials regarding the use of the SGTL2is have been conducted. Most of them suggest that the use of empagliflozin, canagliflozin, and dapagliflozin in patients with kidney disease is associated with a reduced risk of sustained-kidney-function loss and a reduction in the albuminuria, and attenuates the eGFR decline and a reduction in the risks of major adverse kidney events and all-cause mortality in patients with or without diabetic kidney disease. All this evidence supports the renoprotective role of the SGLT2i [29,89,90,91].

## 7. Interstitial Volume

In an average human body, 60% of the total body weight consists of water. This can be categorized as intracellular and extracellular fluid volume. The extracellular fluid volume constitutes about one-third of the total fluid volume in the human body and can further be divided into the interstitial fluid volume and the plasma. The interstitial fluid volume constitutes about 75% of the extracellular fluid and consists of fluid that surrounds the cells in the tissues of the body, serving as a crucial component of the extracellular matrix [92].

In conditions such as HF and kidney dysfunction, there is a disturbance in fluid management. These conditions are linked to a significantly increasing volume of the interstitial fluid. More specifically, in HF, the incompetence of the heart leads to an activation of the neurohormonal system of renin–angiotensin–aldosterone, which can lead to severe sodium and fluid retention [93]. In addition, renal failure is a clinical condition in which the kidneys’ ability to filter and excrete excess fluids is impaired, causing an increase in the interstitial volume as well [94]. In that way, the fluid overload leads to hemodynamic congestion with increased central filling pressures and the clinical manifestation of congestion [93].

SGLT2is have been shown to effectively reduce the interstitial fluid volume. The inhibition of the SGLT2 reduces glucose and sodium reabsorption in the proximal tubule, which reduces the osmotic gradient between the distal tubular fluid and the interstitial and results in osmotic diuresis [95]. Many studies support that the use of SGLT2is through that mechanism reduces extracellular volume, ameliorates fluid detention, and tends to maintain an euvolemic fluid status for the short term, up to 24 months [96,97,98,99]. However, more research in that field is needed to ascertain if they can maintain euvolemic status in the long term.

## 8. Gut Microbiota

In general, the term gut microbiota refers to a diverse community of microorganisms that coexist in the human body. Many of them reside in the human body since birth in the gastrointestinal tract. These microorganisms consist of bacteria, fungi, and viruses. They have many utilities such as the synthesis of essential ingredients, digestion, as well as the influence on the immune system [100].

However, the composition and their balance depend on many factors and differ from one human being to another. Recently, the gut microbiota have been associated with many metabolic diseases, such as type II diabetes mellitus, obesity, non-alcoholic fatty liver disease (NAFLD), and cardiovascular diseases [101,102,103]. More specifically, regarding the cardiovascular conditions, the microbiota demonstrate a significant role in CVD prevention. Their effects consist of the regulation of blood pressure, the reduction in inflammation, and the altering of the oxidation rate [104,105]. Among the other pleiotropic actions of the SGLT2is, there seems to be a favorable effect on microbiota in patients with diabetes mellitus type II, cardiovascular disease, and obesity in studies on mice [23]. In human studies, the results seem controversial so far; adding dapagliflozin to metformin does not seem to alter the microbiota composition. However, metformin itself alters the composition of the microbiota; therefore, it was suggested that there may be some overlap between those two [106,107,108]. Moreover, in another study, empagliflozin improved cardiovascular risk factors and glucose control with an increase in short-chain-fatty-acid-releasing bacteria (SCFA-releasing) when compared to metformin. The latter suggests that there may be some effect of the empagliflozin on the gut microbiota itself [109]. In another human study, the use of canagliflozin seems to lead to an increase in the relative abundance of SCFA-releasing bacteria, such as Bacteroides and Lachnospiraceae UCG 004 and NK4A136 group. The suggestion of this study is that the use of canagliflozin regulates glucose control with a potential contribution to the restoration of balance in the gut microbiota as well [110].

## 9. Clinical Implications

Over the past decade, SGLT2is have demonstrated significant benefits beyond their use in diabetes management. Large randomized controlled trials have shown that, in addition to standard medical care, SGLT2is improve outcomes in patients with HFrEF by reducing morbidity and mortality, lowering hospitalization rates, and alleviating congestion symptoms [25,111]. Furthermore, these benefits extend to patients with HF with mildly reduced or preserved ejection fraction, where there has traditionally been no established treatment for improving prognosis [31,112]. In addition to their cardiovascular benefits and decongestion effects, SGLT2is provide renal protection and preserve kidney function, slowing the progression of kidney damage and reducing the need for renal replacement therapy [25,30,31,111,112]. Reflecting these findings, the latest European Society of Cardiology (ESC) guidelines recommend the use of SGLT2is across all HF classes, except for patients with type 1 diabetes or those with advanced renal impairment and a glomerular filtration rate (GFR) below 30 or even 20 mL/min/1.73 m^2^ [3]. Importantly, unlike other first-line treatments for HF, SGLT2is, alongside diuretics, are recommended across all HF categories based on LVEF. Moreover, SGLT2is not only provide symptomatic relief but also may improve survival outcomes [31,112]. Additionally, SGLT2is are safe for use in acute HF or following acute myocardial infarction. They have a neutral hemodynamic effect, do not require dose up-titration, and may facilitate quicker achievement of target doses [113,114].

## 10. Conclusions

SGLT2 inhibitors represent a significant advancement in the treatment of HF due to their multifaceted mechanisms that extend beyond glucose regulation. These inhibitors improve myocardial energy efficiency, reduce oxidative stress, and modulate inflammatory pathways, contributing to cardioprotection and improved cardiac function. Additionally, SGLT2 inhibitors promote autophagy, aiding in the removal of damaged cellular components and preserving myocardial cell integrity. Their beneficial effects also extend to kidney function, where they slow the progression of chronic kidney disease CKD by reducing glomerular pressure and albuminuria. Beyond the multiple reported mechanisms of SGLT2 inhibitors in HF, further research can fully elucidate their pleiotropic effects and optimize their clinical application.

## Figures and Tables

**Table 1:** Pleiotropic effects of SGLT2is in preclinical studies.

Model	Type	SGLTi	Findings	Result	References
Cardiomyocytes of mice	In vivo + In vitro	Empagliflozin	Activation of AMPK ↑M2 marker proteins Improved AMPK phosphorylation and ATP/ADP ↓Cardiac iNOS ↓plasma TNFa +CKMB	Reduced inflammation	[14]
Mice and adipocytes	In vivo + In vitro	Canagliflozin	Mitochondrial biogenesis via AMPK-Sirt1-Pgc1a pathway ↑expression of Sirt1 and Pgc-1α. ↑Mitochondrial oxidative phosphorylation, fatty acid oxidation and thermogenesis	Regulation energy homoeostasis	[15]
Mice	In vivo	Empagliflozin	↑Sestrin2 levels ↑AMPK and eNOS phosphorylation Inhibition of mTOR phosphorylation Improved myocardial hypertrophy/fibrosis ↓cardiac fat accumulation and mitochondrial injury	Antioxidant and anti-inflammatory activity	[16]
Mice	In vivo	Empagliflozin	↑mitochondrial biogenesis ↓ROS production ↑endogenous antioxidants ↑autophagy ↓cardiac apoptosis	Enhance mitochondrial function. Improve cardiac function and remodeling.	[17]
Mice	In vivo	Empagliflozin	inhibition of the transforming growth factor β/Smad pathway Activation of Nrf2/ARE signaling	Ameliorated myocardial oxidative stress injury and cardiac fibrosis	[18]
Mice	In vivo	Dapagliflozin	↓NLRP3 ↓TNFα ↓interleukin-6 ↑AMPK activated mTOR ↑RICTOR levels ↓BNP and Caspase-1 mRNA levels	Reduced inflammation. Improvements in LVESV, LVEDV, LVEF	[19]
RAW 264.7 macrophages	In vitro	Empagliflozin	↓PGE_2,_ COX-2,iNOS ↓Proinflammatory cytokines and chemokines Blocked NF-*κ*B, JNK, and STAT1/3 phosphorylation	Anti-inflammatory effects	[20]
Rats	In vivo	Empagliflozin	↑ Nrf2, LC3-II/LC3-I and bcl2/bax ratios ↓TNFa, IL-1β, MDA	Reduction in oxidative stress, inflammation, and apoptosis. Improved renal I/R injury.	[21]
Mice	In vivo	Dapagliflozin	↓IL-1β, cardiac caspase-1 activity ↓intracellular Ca^2+^ and Na^+^ in cardiomyocytes ↓Serum levels of cTnI, CK-MB, and LDH	Mediate autophagy Limit NLRP3 inflammasome activation. Protection against myocardial I/R injury.	[22]
Μice	In vivo	Dapagliflozin	↓MCP-1, IL-1β and IL-6 ↓PWV ↑ EDD +EID	Improvement in arterial stiffness, endothelial dysfunction, and vascular smooth muscle dysfunction. Altered gut microbiota.	[23]

**Abbreviations**: SGLTi, sodium glucose cotransporter inhibitor; AMPK, Adenosine monophosphate-activated protein kinase; iNOS, inducible nitric oxide synthase; TNFa, Tumor necrosis factor alpha; CKMB, creatine kinase myocardial band; Sirt1, silent information regulator; Pgc-1a, peroxisome proliferator-activated receptor γ coactivator-1a;mTOR, mammalian target of rapamycin; ROS, reactive oxygen species; Nrf2/ARE, Nuclear factor erythroid 2-like 2; NLRP3, NOD-like receptor 3; BNP, brain natriuretic peptide; LVEF, left ventricular ejection fraction; LVESV, left ventricular end-systolic volume; LVEDV, left ventricular end-diastolic volume; PGE2, prostaglandin E_2_;COX-2, Cyclooxygenase-2; NF-*κ*B, Nuclear factor kappa-light-chain-enhancer of activated B cells; JNK, Jun N-terminal kinase; MDA, malondialdehyde; I/R, ischemia–reperfusion; LDH, Lactate dehydrogenase; cTnI, cardiac Troponin I; PWV, pulse wave velocity; EDD, endothelial dependent dilatation; EID, endothelial independent dilatation. ↑: increase, ↓: Decrease.

**Table 2:** Effects of SGLT2i in clinical trials.

Trial	Type	Number	SGLT2i	Results	References
EMPA-REG OUTCOME Trial	Randomized double blind	7020	Empagliflozin in DM	↓Cardiovascular death ↓HF hospitalization No difference in MI and stroke ↑Genital infection	[24]
EMPEROR-Preserved Trial	Randomized double blind	5988	EmpagliflozinIn HFpEF	↓Cardiovascular death ↓HF hospitalization ↓Urgent HF visit Improved NYHA status	[4]
EMPEROR-Reduced Trial	Randomized double blind	3730	Empagliflozinin HFrEF	↓Cardiovascular death ↓HF hospitalization ↓Urgent HF visit Improved NYHA status	[5]
DAPA-HF Trial	Randomized double blind	4744	DapagliflozinIn HFrEF	↓Cardiovascular death ↓HF hospitalization ↓Urgent HF visit Improved NYHA status and QOL	[25]
SOLOIST-WHF Trial	Randomized double blind	1222	Sotagliflozin in acute HF and DM	↓Cardiovascular death ↓HF hospitalization ↓Urgent HF visit ↑Diarrhea and Hypoglycemia	[9]
CREDENCE Trial	Randomized double blind	4401	Canagliflozin in DM and CKD	30% lower risk of dialysis, transplantation, or GFR of <15 mL/1.73 m^2^ 34% lower RR of death from renal causes, end stage CKD and doubling creatinine ↓ CVD, MI, Stroke and HF hospitalizations	[26]
EMPA-KIDNEY Trial	Randomized double blind	6609	Empagliflozin in CKD (GFR 20–40 mL/1.73 m^2^)	↓ Progression of CKD and CVD ↓Hospitalization from all causes No change on HF hospitalization and any cause death	[27]
Canvas Programm	Two randomized double blind trials	10,142	Canagliflozin in DM	↓ End stage CKD, death from renal causes ↓albuminuria, eGFR decline ↓ CVD, non fatal MI, stroke and HF hospitalization	[28,29]
DAPA-CKDTrial	Randomized double blind	4304	Dapagliflozin in CKD with and without DM	↓Sustained decline in the estimated GFR of at least 50%, end-stage kidney disease, or death from renal and CVD No difference between DM patients and non	[30]
DELIVER Trial	Randomized double blind	6263	Dapagliflozin in HF and EF > 40%	↓HF hospitalization ↓Urgent HF visit ↓CVD No difference between patients with EF > 60% and <60% No difference between DM patients and non	[31]
EMPA-HEART Trial	Randomized double blind	97	Empagliflozin in DM + CKD + CAD	↓LV mass indexed to BSA ↓Systolic and diastolic BP ↑ Hematocrit	[32]

**Abbreviations**: HF, heart failure; NYHA, New York Heart Association; HFrEF, Heart Failure with Reduced Ejection Fraction; HFpEF, Heart Failure with preserved Ejection Fraction; QOL, Quality Of Life; DM, Diabetes Melitus; CKD, Chronic Kidney Disase; CVD, Cardiovascular Death; RR, Relative Risk; MI, Myocardial Infarction; CAD, Coronary Artery Disease; BSA, Body Surface Area; LV, Left Ventricular; BP, Blood Pressure. ↑: increase, ↓: Decrease.

## List of Abbreviations

AMPK, Adenosine monophosphate-activated protein kinase; ARE, Antioxidant response element; ATP, Adenosine triphosphate; BAX, Bcl-2-associated X protein; BNP, Brain natriuretic peptide; CaMKII, Ca^2+^/calmodulin-dependent protein kinase II; CKD, Chronic kidney disease; CKMB, Creatine kinase myocardial band; COX-2, Cyclooxygenase-2; CRP, C-reactive protein; DAMPs, Damage-associated molecular patterns; DM, Diabetes mellitus; EPO, Erythropoietin; GFR, Glomerular filtration rate; GLUT1, Glucose transporter 1; HF, Heart failure; HFpEF, Heart failure with preserved ejection fraction; HFrEF, Heart failure with reduced ejection fraction; ICAM-1, Intercellular adhesion molecule 1; IL-1, Interleukin 1; IL-6, Interleukin 6; iNOS, Inducible nitric oxide synthase; LDH, Lactate dehydrogenase; LVEF, Left ventricular ejection fraction; mTOR, Mammalian target of rapamycin; NF-κB, Nuclear factor kappa-light-chain-enhancer of activated B cells; NHE, Sodium-hydrogen exchanger; NLRP3, NOD-like receptor 3; Nrf2, Nuclear factor erythroid 2-like 2; NYHA, New York Heart Association; PAMPs, Pathogen associated molecular patterns; PGC-1α, Peroxisome proliferator-activated receptor γ coactivator-1α; PGE2, Prostaglandin E2; PKA, Protein kinase A; PWV, Pulse wave velocity; QOL, Quality of life; ROS, Reactive oxygen species; SGLT2i, Sodium–glucose cotransporter 2 inhibitors; Sirt1, Silent information regulator 1; STAT1, Signal transducer and activator of transcription 1; TLR, Toll-like receptor; TNF-α, Tumor necrosis factor alpha; VCAM-1, Vascular cell adhesion molecule 1.
